# Changes in stroke volume induced by lung recruitment maneuver can predict fluid responsiveness during intraoperative lung-protective ventilation in prone position

**DOI:** 10.1186/s12871-021-01527-y

**Published:** 2021-12-02

**Authors:** Ryota Watanabe, Koichi Suehiro, Akira Mukai, Katsuaki Tanaka, Tokuhiro Yamada, Takashi Mori, Kiyonobu Nishikawa

**Affiliations:** Department of Anesthesiology, Osaka City University Graduate School of Medicine, 1-5-7 Asahimachi, Abeno-ku, Osaka, 545-8586 Japan

**Keywords:** Lung recruitment maneuver, Prone position, Fluid responsiveness, Stroke volume

## Abstract

**Background:**

The present study aimed to evaluate the reliability of hemodynamic changes induced by lung recruitment maneuver (LRM) in predicting stroke volume (SV) increase after fluid loading (FL) in prone position.

**Methods:**

Thirty patients undergoing spine surgery in prone position were enrolled. Lung-protective ventilation (tidal volume, 6–7 mL/kg; positive end-expiratory pressure, 5 cmH_2_O) was provided to all patients. LRM (30 cmH_2_O for 30 s) was performed. Hemodynamic variables including mean arterial pressure (MAP), heart rate, SV, SV variation (SVV), and pulse pressure variation (PPV) were simultaneously recorded before, during, and at 5 min after LRM and after FL (250 mL in 10 min). Receiver operating characteristic curves were generated to evaluate the predictability of SVV, PPV, and SV decrease by LRM (ΔSV_LRM_) for SV responders (SV increase after FL > 10%). The gray zone approach was applied for ΔSV_LRM_.

**Results:**

Areas under the curve (AUCs) for ΔSV_LRM_, SVV, and PPV to predict SV responders were 0.778 (95% confidence interval: 0.590–0.909), 0.563 (0.371–0.743), and 0.502 (0.315–0.689), respectively. The optimal threshold for ΔSV_LRM_ was 30% (sensitivity, 92.3%; specificity, 70.6%). With the gray zone approach, the inconclusive values ranged 25 to 75% for ΔSV_LRM_ (including 50% of enrolled patients).

**Conclusion:**

In prone position, LRM-induced SV decrease predicted SV increase after FL with higher reliability than traditional dynamic indices. On the other hand, considering the relatively large gray zone in this study, future research is needed to further improve the clinical significance.

**Trial registration:**

UMIN Clinical Trial Registry UMIN000027966. Registered 28th June 2017.

## Background

Perioperative fluid therapy is a routine part of clinical practice for most anesthesiologists, but there are several challenges. One of the most complex aspects of perioperative fluid therapy is determining how much fluid should be administered in each patient. Optimal fluid management improves perioperative outcomes, [1, 2] while excessive or inadequate infusion can increase the morbidity [3, 4]. Therefore, it is an issue of great importance to find an appropriate parameter to predict fluid responsiveness [5]. Static indices, including central venous pressure, pulmonary capillary wedge pressure, and global end-diastolic volume index, have traditionally been used for volume assessment but are of limited value in predicting fluid responsiveness [6]. In contrast, dynamic indices, such as stroke volume variation (SVV) and pulse pressure variation (PPV), have recently been used and are superior to static indices in discriminating fluid responders [7, 8]. However, the reliability of these indices is below the clinically acceptable level in patients with low tidal volume (less than 8 mL/kg of body weight) and in those with low airway driving pressure (less than 20 cm H_2_O) [9, 10].

Lung-protective ventilation, defined as lower tidal volume (6–7 mL/kg) and positive end-expiratory pressure (PEEP) (5–10 cm H_2_O), is becoming a standard of care for surgical patients and can improve the outcomes of postoperative patients [11, 12]. In this situation, the usefulness of dynamic indices in predicting fluid responsiveness is limited, reducing their applicability in daily clinical practice [13]. Lung recruitment maneuver (LRM) is a fundamental technique in lung-protective ventilation to reopen the lung alveoli. Furthermore, LRM induces hemodynamic changes including a transient decrease in venous return and stroke volume (SV). A previous study showed that LRM-induced SV changes can predict SV increase after volume expansion, even in patients undergoing lung-protective ventilation [14]. However, the usefulness of LRM in assessing volume status in prone position has not been evaluated. Prone positioning, which is often employed in neurosurgical surgery, represents a challenging setting under this circumstance owing to the possibility of massive bleeding [15]. In addition, prone positioning is associated with SV reduction induced by vena caval compression and increased intrathoracic pressure [16]. Therefore, identification of fluid responders with respect to SV in this setting is a major concern for anesthesiologists. Lung-protective ventilation is commonly used in prone position; thus, under this condition, the usefulness of dynamic indices is limited, as described in a previous report [16].

The present study aimed (1) to evaluate the reliability of LRM-induced hemodynamic changes in predicting SV increase after fluid loading in prone position and (2) to compare the predictability of this indicator for fluid responsiveness with that of traditional dynamic indices in patients undergoing lung-protective ventilation in prone position.

## Methods

### Anesthetic management

Ethical approval was given by the Ethical Committee of Osaka City University Graduate School of Medicine, (No. 3693, Chairperson Prof. Tetsuo Arakawa) on 23 February 2017. The current study was registered on the UMIN Clinical Trials Registry database (registration number: UMIN000027966) before the initial enrolment. Written informed consent was obtained from all patients. In this study, patients undergoing spine surgery in prone position were enrolled. The exclusion criteria were as follows: those younger than 20 years and those who had atrial fibrillation, symptomatic cerebrovascular disease, and/or reduced cardiac function (ejection fraction < 50%).

No premedication was provided. Each patient consumed clear liquids until 3 h before surgery. Anesthesia was induced with propofol, remifentanil, and rocuronium. After securing the airway, lung-protective mechanical ventilation was employed with a tidal volume of 6–7 mL/kg of ideal body weight and PEEP of 5 cm H_2_O. The ventilation rate was adjusted to maintain end-tidal carbon dioxide between 35 and 40 mmHg. General anesthesia was maintained using sevoflurane, desflurane, or propofol and remifentanil. An arterial line was inserted into the radial artery, which was connected to a Vigileo™/FloTrac™ system monitor (Edwards Lifesciences, Irvine, CA, USA). Anesthetic depth was controlled to maintain the bispectral index between 45 and 60 (BIS Vista™ monitoring system; Aspect Medical Systems, Natick, MA, USA). After anesthetic induction, patients were turned to the prone position laying on four pads to relieve pressure to the abdomen.

### Measurement of SV and SVV

SV and SVV were calculated using the Vigileo-FloTrac system, which continuously measures hemodynamic variables by analyzing arterial pressure waveforms from a standard peripheral arterial catheter without the need for external calibration. The mechanism has been described previously in detail [17].

### PPV calculation

Pulse pressure (PP) was defined as the difference between diastolic and systolic arterial pressures. As previously described, [18] PPV was calculated as follows: PPV = (maximum PP – minimum PP) / [(maximum PP + minimum PP) / 2].

### Study protocol

**Fig. 1:**
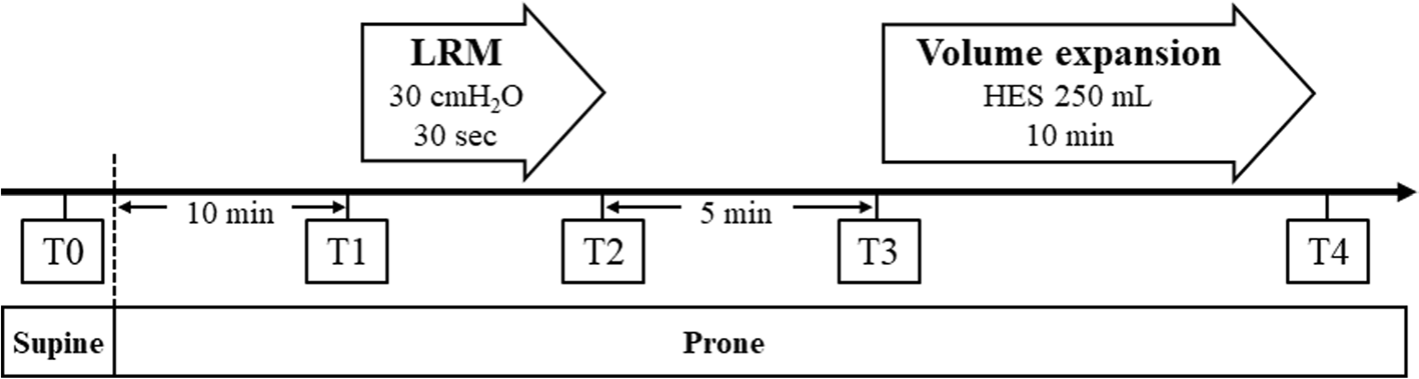
Study protocol. LRM consisted of applying a continuous positive airway pressure of 30 cm H_2_O for 30 s. Volume expansion consisted of an infusion of 250 mL HES given in 10 min. Five sets of hemodynamic measurements including mean arterial pressure, heart rate, stroke volume, stroke volume variation, and pulse pressure variation were performed. T0: baseline measurement in supine position; T1: baseline measurement in prone position 10 min after turning prone and before LRM; T2: at the end of LRM; T3: second baseline measurement 5 min after LRM; T4: after volume expansion. Abbreviations: LRM, lung recruitment maneuver; HES, hydroxyethyl starch

The design of the study is shown in Fig. 1. The study protocol was performed under stable hemodynamic conditions, which was defined as follows: mean arterial pressure (MAP) and heart rate (HR) changes within ±10% in 1 min before measurements [19, 20]. Just before changing position from supine to prone (T0), hemodynamic variables including MAP, HR, SV, SVV, and PPV were recorded (in supine position). Ten minutes after prone positioning, LRM (continuous airway pressure of 30 cmH_2_O for 30 s) was performed, and hemodynamic variables simultaneously recorded before LRM (T1), at the end of LRM (T2), and at 5 min after LRM (T3). The LRM setting in the present study was decided in accordance with a previous study [14]. After recording, volume expansion was performed using 250 mL of Voluven® (hydroxyethyl starch; Otsuka Pharmaceutical Co. Ltd., Tokyo, Japan) in 10 min. After fluid administration (T4), hemodynamic variables were also recorded. SVV and PPV variables at the time points of T0 and T3 were defined as SVV_supine_ / PPV_supine_ (T0), and SVV_prone_ / PPV_prone_ (T3), respectively.

### Statistical analysis

The primary endpoint was set as the reliability of LRM-induced SV change and SVV for fluid responsiveness, with comparison performed using receiver operating characteristic (ROC) analysis. In a previous report, the AUC of ΔSV_LRM_ to discriminate fluid responders in supine position was 0.96 [14]. In a previous study investigating the ability of SVV to discriminate fluid responders in patients undergoing lung-protective ventilation in prone position, the AUC of SVV was reported as 0.53 [16]. Referring to these studies, we hypothesized that AUCs for ΔSV_LRM_ and SVV would be 0.9 and 0.6, respectively. We conducted the power analysis as the following; H0: AUC = 0.6 versus H1: AUC of ΔSV_LRM_ > 0.6 with α = 0.05 and power = 0.80, assuming that the ratio of responders and non-responders would be 1:2. The ratio of responders and non-responders was determined referring to the previous report [21]. According to the power analysis, a sample size was calculated as 27 patients. Considering a drop-out rate of 10%, finally, we planned to include 30 patients in this study.

Correlations between percentage change in SV by LRM (ΔSV_LRM_) and percentage change in SV after fluid loading (ΔSV_FL_) was examined using Pearson correlation coefficient. Furthermore, ΔSV_LRM_ and ΔSV_FL_ were compared using the four-quadrant analysis. In the four-quadrant plot analysis, ΔSV_LRM_ and ΔSV_FL_ were drawn in the four quadrants. The results of this analysis were assessed using the concordance rate, which was defined as the percentage of data points located in the upper right or lower left corner of the four-quadrant plot. The concordance rate is defined as good when it was more than 92%, as shown by Critchley et al. [22] Patients were divided into two groups (responders and nonresponders with respect to SV) according to percentage increases in SV after fluid loading, with *responders* defined as those with ΔSV_FL_ > 10%. The definition of responders was in accordance with previous studies [14, 23]. ROC curves were generated for SVV, PPV, and ΔSV_LRM_ to distinguish responders from nonresponders. In ROC analyses, optimal thresholds and AUCs were calculated. AUCs by ROC analysis were compared using a previously described method [24]. The *p*-values for the ROC analysis were calculated by testing against the hypothesis of AUC = 0.50.

Furthermore, we applied the *gray zone* approach to test the predictive values of ΔSV_LRM_. The gray zone approach was used to assess the values for which the target variables did not provide definitive information, which has been described previously [14, 25]. Briefly, a two-step procedure was employed in the gray zone approach. First, bootstrap resampling was performed for ΔSV_LRM_ and ΔMAP_LRM_. The best cut-off thresholds and their 95% confidence intervals (CI) were calculated from 1000 bootstrapped populations. The optimal threshold was selected to maximize Youden’s index (i.e., J = sensitivity + specificity − 1). Second, the inconclusive range of each value for the assessment of SV or BP responsiveness (i.e., cut-off values with sensitivity < 90% or specificity < 90%) was calculated. If the 95% CI of the optimal threshold from the first step was larger than the inconclusive range from the second step, the values from the first step were obtained as *gray zone* values.

Hemodynamic variables were compared using Student’s paired t-test, Mann–Whitney U test, and chi-squared test. For all analyses, a *p*-value < 0.05 was considered statistically significant. Statistical analysis was performed using StatFlex software version 6.0 (Artech Co. Ltd., Osaka, Japan), and SigmaPlot software version 13.0 (Systat Software Inc., San Jose, CA, USA).

## Results

### Patient characteristics and hemodynamic data

Patient characteristics are summarized in Table 1. A total of 30 patients were enrolled in this study. Hemodynamic variables during the study period are shown in Table 2. There were no complications including prolonged hypotension and fatal arrhythmia during the study. When the patients were turned to the prone position, both of SVV and PPV significantly increased, while SV significantly decreased (T0 vs T1). There were no significant changes in HR and MAP between the time points of T0 and T1. LRM induced a significant decrease in MAP and SV in both groups (T1 vs T2). For responders, SV significantly increased after fluid loading (T3 vs T4).

**Table 1:** Patient characteristics

Variables	All patients (*n* = 30)
Gender, M/F	17/13
Age, yr	59.9 ± 17.9
Height, cm	159 ± 9.8
Weight, kg	59.0 ± 13.6
Body Mass index, kg/m^2^	23.1 ± 4.6
ASA physical status, I/II/III/IV	2/ 23 /4 /1
Comorbidities	
Hypertension, n	13
Coronary artery disease, n	2
Chronic obstructive pulmonary disease, n	1
Asthma, n	1
Chronic kidney disease, n	4
Diabetes, n	5
Dyslipidemia, n	4
Ventilatory conditions	
Tidal volume, ml/kg ideal body weight	6.29 ± 0.47
Respiratory rate, cycles/min	12.1 ± 1.0
Positive endo-expiratory pressure, cm H_2_O	5.0
Peak inspiratory pressure, cm H_2_O	16.7 ± 3.9
End-inspiratory plateau pressure, cm H_2_O	11.3 ± 3.4

Data are expressed as mean (SD) or number unless stated

*Abbreviations*: *ASA* American Society of Anaesthesiologist

**Table 2:** Hemodynamic variables in responders (*n* = 13) and nonresponders (*n* = 17) during the study

Variables	T0	T1	T2	T3	T4	*P* Value T0 versus T1	*P* Value T1 versus T2	*P* Value T3 versus T4
HR (bpm)								
Responders	69 ± 12	68 ± 13	67 ± 13	69 ± 15	68 ± 14	0.428	0.732	0.231
Nonresponders	67 ± 10	66 ± 10	63 ± 10	66 ± 10	66 ± 9	0.496	0.010*	0.744
MAP (mmHg)								
Responders	77 ± 13	77 ± 12	58 ± 13	74 ± 12	80 ± 12	0.801	< 0.001*	0.031*
Nonresponders	78 ± 9	78 ± 9	66 ± 13	78 ± 8	78 ± 8	0.231	< 0.001*	0.859
Stroke volume (mL)								
Responders	62 ± 14	59 ± 14	26 ± 21	56 ± 14	67 ± 15	0.002*	< 0.001*	< 0.001*
Nonresponders	59 ± 11	58 ± 11	39 ± 17	58 ± 11	60 ± 11	0.009*	< 0.001*	0.002*
SVV (%)								
Responders	10 ± 3	12 ± 4		12 ± 4	10 ± 2	0.005*		0.047*
Nonresponders	12 ± 4	13 ± 6		13 ± 5	12 ± 5	0.046*		0.067
PPV (%)								
Responders	9 ± 3	10 ± 4		10 ± 5	9 ± 3	0.015*		0.057
Nonresponders	10 ± 6	11 ± 6		11 ± 5	9 ± 5	0.037*		0.008*

Statistical analysis Paired t-test. Data are expressed as mean ± SD. * statistically significant *p* < 0.05

*Abbreviations*: *BP* Blood pressure, *HR* Heart rate, *MAP* Mean arterial blood pressure, *SVV* Stroke volume variation, *PPV* Pulse Pressure variation, T0, just before changing position from supine to prone; T1, before recruitment maneuver in prone position; T2, at the end of recruitment maneuver; T3, 5 min after recruitment maneuver / baseline before volume expansion; T4, after volume expansion

### SV changes during the study period

**Fig. 2:**
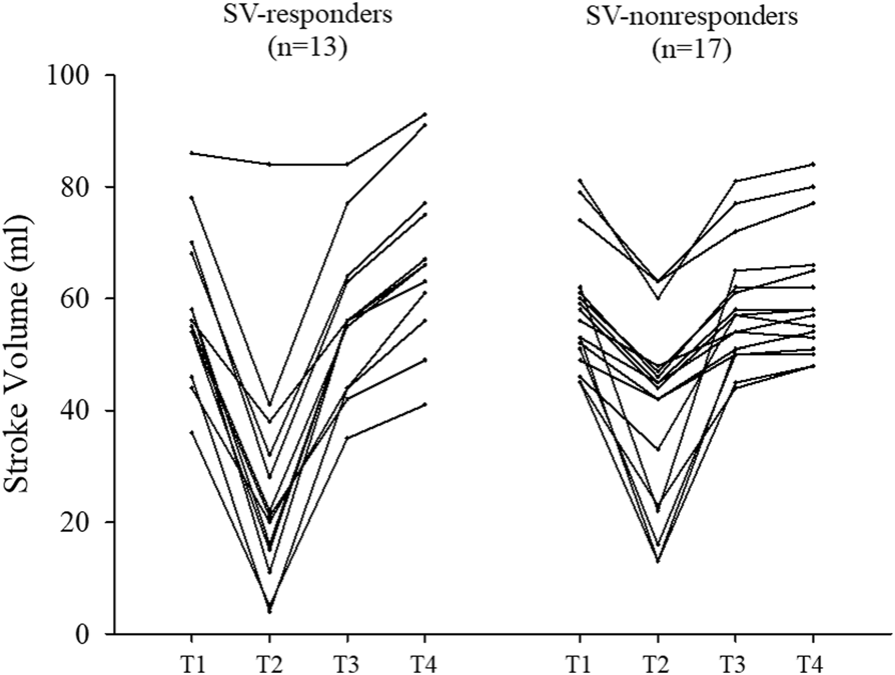
Change in among responders and nonresponders to fluid loading at points T1 (before lung recruitment maneuver), T2 (at the end of lung recruitment maneuver), T3 (at 5 min after lung recruitment maneuver), and T4 (after volume expansion). SV, stroke volume

**Fig. 3:**
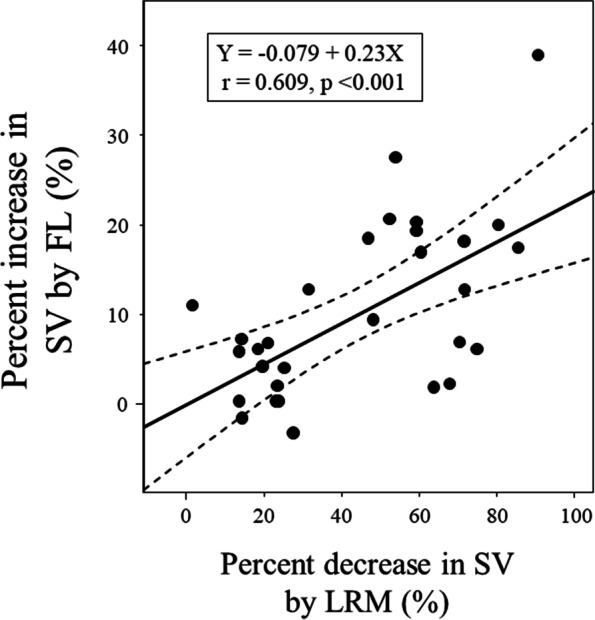
Correlations between percentage change in SV after FL and percentage decrease in SV by LRM. SV, stroke volume; FL, fluid loading; LRM, lung recruitment maneuver

**Fig. 4:**
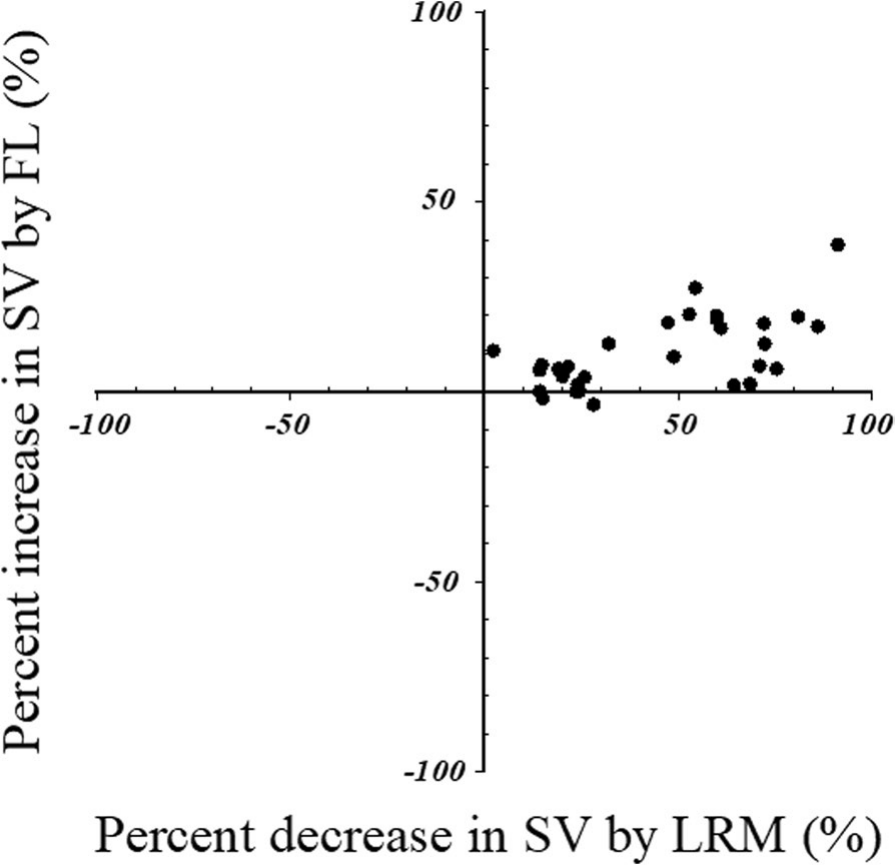
Four quadrant plot analysis to examine the concordance between percentage change in SV after FL and percentage decrease in SV by LRM. SV, stroke volume; FL, fluid loading; LRM, lung recruitment maneuver

There were 13 fluid responders with respect to SV. Figure 2 shows the SV change in responders and nonresponders during the study period. The SV decrease during LRM was greater in responders (33 ± 12 mL) than in nonresponders (19 ± 11 mL) (*p* = 0.004). As shown in Fig. 3, ΔSV_LRM_ were significantly correlated with ΔSV_FL_ (ΔSV_LRM_: r = 0.609, *p* <  0.001). The trending ability of ΔSV_LRM_ was examined using four-quadrant analysis (Fig. 4). In the four-quadrant analysis, the concordance rate was 93.3%.

### Predictability of fluid responsiveness with respect to SV

**Fig. 5:**
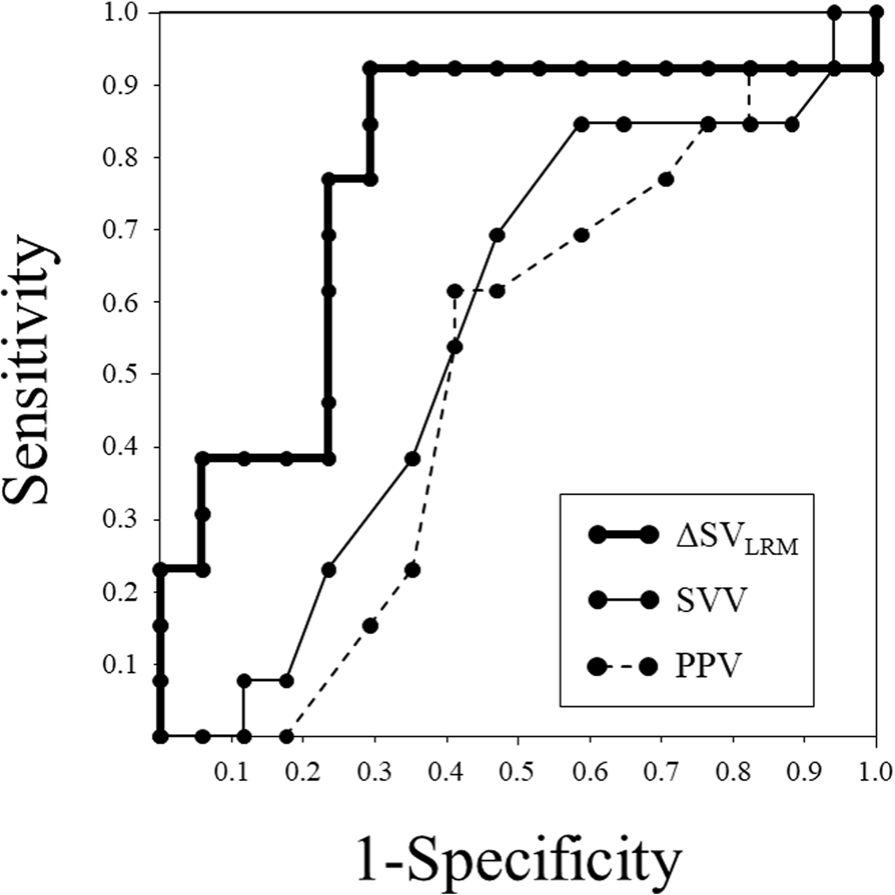
Receiver operating characteristic curves for SVV_prone_, PPV_prone_, and ΔSV_LRM_ to distinguish responders (SV increase after fluid loading > 10%). SV, stroke volume; BP, blood pressure; SVV_prone_, stroke volume variation at time point T3; PPV_prone_, pulse pressure variation at time point T3; ΔSV_LRM_, decrease in stroke volume by lung recruitment maneuver

Figure 5 illustrates the ROC analysis for the assessment of the ability to discriminate fluid responders with respect to SV. The AUCs for ΔSV_LRM_, SVV_prone_, and PPV_prone_ to predict responders were 0.778 (95% CI: 0.590–0.909), 0.563 (95% CI: 0.371–0.743), and 0.502 (95% CI: 0.315–0.689), respectively. ΔSV_LRM_ showed the best predictability in responders (*p* = 0.003), whereas both SVV_prone_ and PPV_prone_ were not significant predictors in responders (*p* = 0.563 and 0.984, respectively). The optimal threshold for ΔSV_LRM_ was 30%, with a sensitivity of 92.3% and a specificity of 70.6%. Furthermore, SVV_supine_ and PPV_supine_ were not significant predictors for SV-responsiveness (SVV_supine_, AUC: 0.611, *p* = 0.293; PPV_supine_, AUC: 0.550, *p* = 0.646) (Table 3).

**Table 3:** ROC analyses for ΔSV_LRM_, SVV_prone_, PPV_prone_, SVV_supine_ and PPV_spine_ to discriminate responders

Variables	Cut-off value (%)	AUC (95%CI)	Sensitivity (%)	Specificity (%)	*P* value
ΔSV_LRM_ (%)	30	0.778 (0.590–0.909)	92.3	70.6	0.003*
SVV_prone_ (%)	13	0.563 (0.371–0.743)	84.6	41.2	0.563
PPV_prone_ (%)	9	0.502 (0.315–0.689)	61.5	58.8	0.984
SVV_supine_ (%)	8	0.611 (0.417–0.782)	38.5	82.4	0.293
PPV_supine_ (%)	10	0.550 (0.358–0.731)	84.6	35.3	0.646

*statistically significant *p* < 0.05

*Abbreviations*: *ROC* Receiver operating characteristics, *SV* Stroke volume, *ΔSV*_*LRM*_ Decrease in stroke volume by lung recruitment maneuver, *SVV*_*prone*_ Stroke volume variation at time point T3, *PPV*_*prone*_ Pulse pressure variation at time point T3, *SVV*_*supine*_ Stroke volume variation at time point T0, *PPV*_*spine*_ Pulse pressure variation at time point T0, *AUC* Area under the curve

**Fig. 6:**
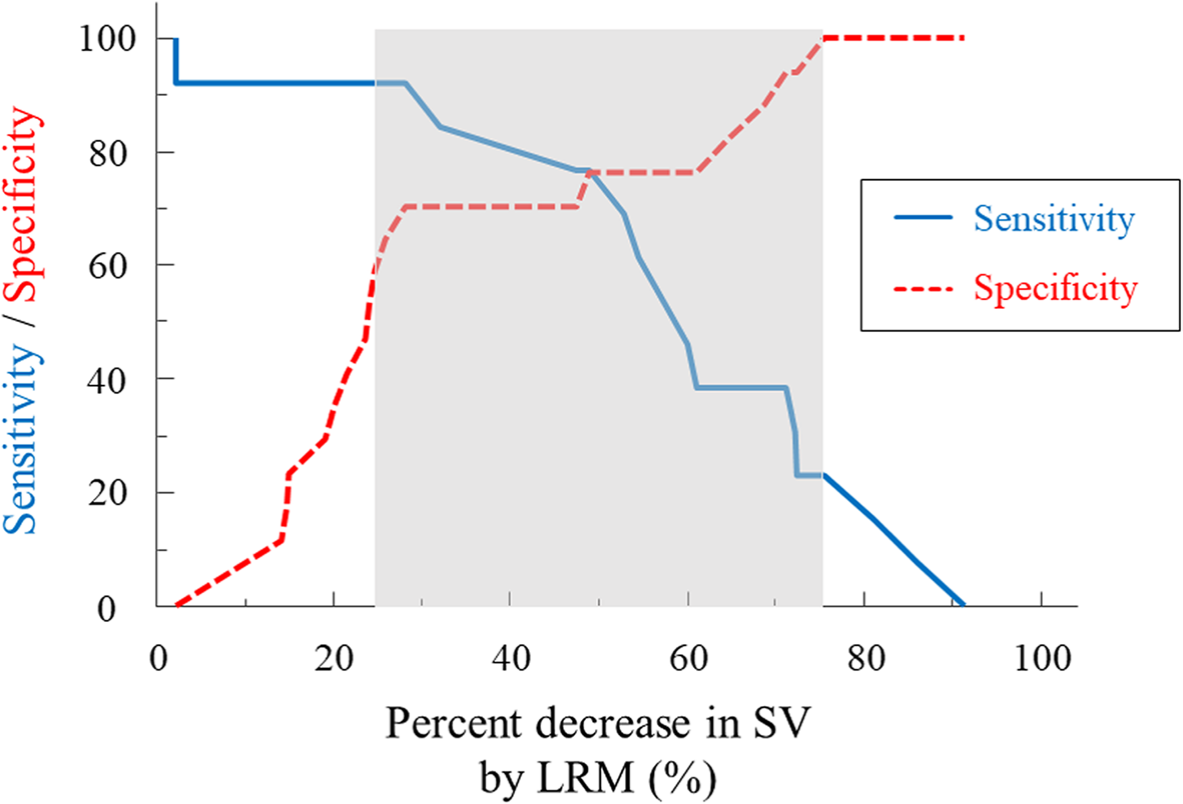
Gray zone for ΔSV_LRM_. Blue and red lines denote sensitivity and specificity, respectively. The gray zone indicates the inconclusive range for each variable. ΔSV_LRM_, decrease in stroke volume by lung recruitment maneuver; SV, stroke volume; LRM, lung recruitment maneuver

With the gray zone approach, the inconclusive range of ΔSV_LRM_ for SV responders was 25–75% (including 50% of enrolled patients; Fig. 6).

## Discussion

In the present study, we investigated the reliability of LRM-induced hemodynamic changes in predicting SV response after fluid administration in patients undergoing lung-protective ventilation in prone position. The SV decrease (30%) during LRM could predict SV increase after fluid administration. ΔSV_LRM_ was significantly correlated with ΔSV_FL_. The predictability of ΔSV_LRM_ for fluid responsiveness was better than that of SVV and PPV, which are traditional indicators of fluid responsiveness.

Prone positioning is commonly employed during various surgical procedures, especially brain and spine surgeries, and induces a decrease in chest compliance and an increase in abdominal pressure, which in turn affects hemodynamics such as decreased venous return and stroke volume. Volume expansion is the first-line treatment for CO reduction during surgery, [26] but inadequate fluid loading in prone position can lead to an increase in edema of the larynx and pharynx, making extubation difficult in the operating room [27, 28]. On the other hand, intraoperative goal-directed fluid therapy based on SV optimization improves postoperative outcomes in surgical patients [29]. Hence, predicting fluid responsiveness in prone position is an issue of major concern among anesthesiologists.

Previous studies [16, 18, 30, 31] investigating the reliability of dynamic indices in assessing fluid responsiveness in prone position have presented various results. Even in the supine position, the reliability of dynamic indices is limited in patients with low tidal volume or low ventilatory driving pressure. In fact, in the study by Min et al. without lung-protective ventilation, SVV served as a good predictor of fluid responsiveness in prone position, with an AUC of 0.78 in the ROC analysis [31]. Conversely, the predictability of SVV became poorer among patients undergoing lung-protective ventilation in prone position, with an AUC of 0.53 [16]. Yonis et al. also showed that 100% of the patients with lung-protective ventilation in prone position were ​in the gray zone for PPV [32]. Lung-protective ventilation is becoming a standard for intraoperative management and for improving postoperative outcomes during in prone position [33]. Considering the increasing application of lung-protective ventilation in the surgical population, [34] the applicability of dynamic indices in prone position would diminish. Therefore, a new approach for the assessment of fluid responders in prone position is required.

Intraoperative LRM is often used as part of lung protective ventilation to reduce intraoperative lung collapse, improve oxygenation, and reduce the incidence of postoperative pulmonary complications (PPC) [35]. Xiong et al. showed that intraoperative lung protective ventilation including LRM prevents PPC also in prone spine surgery [33]. LRM increases intrathoracic and transpulmonary pressures, which results in decreased venous return and increased pulmonary vascular resistance, thus leading to decreased SV [14]. LRM-induced hemodynamic collapse depends on volume status. In an experimental study, [36] volume depletion by LRM was significantly higher under hypovolemic condition than under normo- and hypervolemic conditions. Taking this into account, Biais et al. [14] applied LRM for the assessment of fluid responsiveness. In their clinical study, a 30% decrease in SV during LRM could predict SV increase after volume expansion, with a sensitivity of 88% and a specificity of 92%. In the present study, we investigated the ability of LRM-induced hemodynamic changes to predict fluid responsiveness in prone position. Consistent with the results of the study by Biais et al., [14] even in prone position, a 30% decrease in ΔSV_LRM_ could predict a 10% increase in ΔSV_FL_ with high sensitivity and specificity (92.3 and 70.6%, respectively). On the other hand, the traditional measures of SVV and PPV were not significant discriminators, which is consistent with previous studies. As shown in our study, the predictability of ΔSV_LRM_ for fluid responsiveness was better than that of SVV and PPV among patients in prone position.

Although there were no complications induced by LRM in this study, there is a risk of adverse events such as severe hypotension, fatal arrhythmia, and pneumothorax with LRM procedure [37, 38]. In the current study, the decrease in MAP induced by LRM (responders: 77 to 58 mmHg, nonresponders: 78 to 66 mmHg) were similar with those in the previous study [14] (LRM was performed in supine position; responders: 71 to 51 mmHg, nonresponders: 71 to 55 mmHg). As indicated by Young et al., [39] LRM should be performed when the patients’ oxygen saturation is constantly low (less than 94%) and following a disconnection from the respiratory circuit. LRM should be considered according to an individual risk-benefit assessment and should not be routinely applied for assessing fluid responsiveness. Anesthesiologists can perform LRM procedures to assess fluid responsiveness when dynamic indices such as SVV and PPV are within the gray zone. In addition, considering the adverse effects induced by LRM, future studies are needed to decide whether ΔSV_LRM_ in shorter duration and/or at lower pressure can maintain a high accuracy in discriminating fluid responders.

In this study, the percentage of fluid responders was 43%, which is lower than that (approximately 50%) indicated in several systematic reviews about fluid responsiveness [40, 41]. This difference may be due to the differences in the amount of fluid infused and the definition of fluid responders.

We applied the gray zone approach to determine the inconclusive range for ΔSV_LRM_. The inconclusive range for ΔSV_LRM_ was 25–75% (including 50% of all patients). The inconclusive range was relatively larger for ΔSV_LRM_ than that reported in a previous study investigating it in spine position (22–37%, including 36% of enrolled patients). This discrepancy may be due to the large distribution of ΔSV_LRM_ (2–91%) in our study. Prone positioning reduces chest compliance, the change of which depends on patients’ body constitution. Various changes in chest compliance may lead to a wide range of hemodynamic effects during LRM. Although the predictability of ΔSV_LRM_ for fluid responsiveness was excellent in the ROC analyses, considering the gray zone, these indices should be carefully used in clinical practice.

Our study has some limitations. First, we used the Vigileo/FloTrac system to measure SV. The accuracy of this system in measuring SV depends on systemic vascular resistance, [17] which can be a major limitation. Second, the sample size of this study might be inadequate. In this study, we conducted the power analysis using a difference of 0.90 and 0.60, referring to previous studies. However, if the AUC of 0.75, which is the threshold for considering a diagnostic test to be accurate [42], was used as the null hypothesis, the sample size would be larger. It is also possible that an increase in the number of enrolled patients would narrow the inconclusive range of ΔSV_LRM_. Third, the duration of hemodynamic stability of 1 min might be short. However, we decided the definition referred to the previous studies assessing fluid responsiveness [19, 20]. Additionally, as shown in Table 2, HR and MAP at T0, T1 and T3 were quite similar. Therefore, the impact of this limitation on the results is minimized. Fourth, intraoperative use of HES may lead to the incidence of adverse outcomes in abdominal surgery [43]. However, in this study, we administered 250 mL of HES solely to confirm fluid responsiveness, and do not recommend the continuous administration of HES during surgery. The administration of HES (250 mL) are frequently used in previous studies assessing fluid responsiveness, [19, 44] and we consider that there may be few adverse effects induced by this infusion. Despite these limitations, the present study showed a new approach for assessing hemodynamic response after volume expansion in prone position.

## Conclusions

In conclusion, in patients undergoing lung-protective ventilation in prone position, LRM-induced SV decrease predicted SV increase after fluid loading with a higher reliability than traditional dynamic indices, including SVV and PPV. On the other hand, considering that ΔSV_LRM_ had a relatively large gray zone and there is no consensus on which setting of LRM is particularly effective in predicting fluid responsiveness, it should be further investigated whether ΔSV_LRM_ in different settings can provide a higher accuracy in discriminating fluid responders. As intraoperative hypervolemia can lead to an increase in postoperative complications, volume loading should be carefully performed according to the need of patients [45]. Routine volume loading should not be given solely based on the presence of fluid responsiveness [45]. Further studies are required to construct a new protocol for goal-directed fluid therapy that takes LRM-induced hemodynamic changes into account.

## Data Availability

The datasets analysed during the current study are available from the corresponding author on reasonable request.

## Abbreviations

BP: Blood pressure
CO: Cardiac output
SVV: Stroke volume variation
PPV: Pulse pressure variation
PEEP: Positive end-expiratory pressure
LRM: Lung recruitment maneuver
SV: Stroke volume
HR: Heart rate
MAP: Mean arterial pressure
FL: Fluid loading
ROC: Receiver operating characteristic
AUC: Areas under the curve
CI: Confidence intervals
ASA: American Society of Anaesthesiologist
